# Pharmabiotics as an Emerging Medication for Metabolic Syndrome and Its Related Diseases

**DOI:** 10.3390/molecules22101795

**Published:** 2017-10-24

**Authors:** Thi Thanh Binh Nguyen, Yan Yan Jin, Hea-Jong Chung, Seong-Tschool Hong

**Affiliations:** 1 Department of Biomedical Sciences and Institute for Medical Science, Chonbuk National University Medical School, Jeonju, Chonbuk 54907, Korea; binhpc2311@gmail.com (T.T.B.N.); kimyanyan@jbnu.ac.kr (Y.Y.J.); 2 Department of Microbiology, Seonam University Medical School, Namwon, Chonbuk 55321, Korea; hjchung@jbnu.ac.kr

**Keywords:** pharmabiotics, metabolic syndrome, obesity, diabetes, dyslipidemia, hypertension, cardiovascular diseases, gut microbiota

## Abstract

Metabolic syndrome (MetS) is a cluster of metabolic risk factors associated with central obesity, hyperglycemia, insulin resistance, dyslipidemia and high blood pressure. In recent decades, because of the remarkable increase in both prevalence and severity, MetS and its related diseases such as cardiovascular diseases (CVDs), obesity, hypertension and diabetes have become the main global burden and challenge in strategic management involving prevention and treatment. However, currently, the preventions and treatments based on pharmaceutical interventions do not provide a solution for MetS and its related diseases. Recently, gut microbiota showed clear evidence of preventing and/or treating MetS, shedding light on treating MetS and its related diseases through a completely different approach. In this review, we will interpret the effects of current pharmaceutical drugs used in preventing and treating MetS and its related diseases to understand remaining issues of those interventions. We will explore the possibility of developing gut microbiota as pharmabiotics in a completely new medication option for treating MetS and its related diseases.

## 1. Introduction

Advances in modern medicine including successful immunization programs and new generation antibiotics have effectively controlled infectious diseases in recent decades; however, non-infectious diseases have become a vexing issue sweeping the whole world instead [1], in which metabolic syndrome (MetS) and its related diseases such as CVDs, obesity, hypertension, and diabetes are the most common types of non-infectious diseases threatening the health of mankind [2,3]. In fact, MetS is a complicated condition as presented by its definition and its utility [4,5]. Currently, there is no unanimous definition, and therefore, the most common definition and criteria of MetS from the World Health Organization (WHO), the International Diabetes Federation (IDF), the National Cholesterol Education Program Adult Treatment Panel III (NCEP ATP III) or the American Heart Association (AHA) are widely used at the same time [5]. In general, MetS is a cluster of metabolic risk factors associated with central obesity, hyperglycemia, insulin resistance, dyslipidemia and high blood pressure [2]. The remarkable increase in both the prevalence and severity of MetS noted in all reports of health organizations recently revealed that MetS is the main global burden and challenge in strategic management involving prevention and treatment [6,7,8]. As one of the most significant contributors in MetS, obesity is a major health problem due to its increasing prevalence [9]. Between 1980 and 2015, the prevalence of being overweight worldwide had more than doubled, representing more than 1.9 billion overweight and around 600 billion obese adults [10]. It has been estimated that by 2025, nearly 1.6 billion of the global population will be obese if the current rate is maintained. On the other hand, the prevalence of diabetes and CVDs has been increasing more rapidly in recent decades [11,12]. According to the WHO estimates, over the last 30 years, the diabetes rate had increased more than two-fold since 1980, and in 2013, 422 million adults with diabetes were reported, with the global prevalence being about 8.5% [13]. It is also predicted that the diabetes population may reach to an estimated 5.4% of the population by the year 2025 [14]. Because MetS increases the risks of cardiovascular risk factors including reduced high-density lipoprotein cholesterol (HDL-C), elevated triglycerides (TG) and increased blood pressure, global atherosclerotic CVDs are rapidly increasing in prevalence worldwide [15,16,17]. Furthermore, MetS-related diseases represent the leading causes of death and morbidity in the world [6]. According to the WHO Annual Report, CVDs and diabetes are already in the top ten causes of death in countries worldwide, but with increasing trends in developing countries that currently account for 80% of the world’s population [6,18]. In 2014, an estimated 1.3 million deaths and 1.7 million deaths were directly caused by diabetes and CVDs, respectively [6]. Indeed, with a substantially high annual prevalence of increasing its related diseases, MetS is a matter of great concern [19]. MetS in developing countries, as well as in adolescents and children shows a rapidly increasing trend due to the economic development and nutrition modification [20,21,22,23,24]. Analyzing data from 144 countries in cross-sectional surveys, over 43 million children were estimated to be overweight and obese [20]. Within the last two decades, the worldwide prevalence of children being overweight elevated from 4.2% in 1990 to 6.7% in 2010 and is predicted to reach 9.1% in 2020 [20]. Notably, about more than 80% of children being overweight was observed in developing countries, especially in Africa and Asia, where undernutrition problems have been an unsolved burden [20,25].

It has been well known that MetS is a multifactorial health condition involved genetic, physiological, behavioral and environmental factors such as the lack of physical activities and high caloric food intake [2]. These factors lead to significant chronic alterations in body composition with accumulating excessive abdominal adiposity, increasing body mass index (BMI), glucose or lipid metabolism and, finally, changing the host metabolic phenotype. While the factors belong to physical health are found early, the implicit changes in mental health have rarely been recognized [26,27]. Therefore, the treatment for MetS to control numerous biochemical indexes is a serious challenge in clinical practice for all physicians. Current management of MetS and its related diseases has depended on pharmaceutical interventions [28]. However, besides the short-term benefits, medications are reported for their limited efficacy, as well as serious side effects in the long-term duration of treatment [29,30,31]. Furthermore, the prolonged treatment or the combination of several drugs to treat these MetS and its related diseases may increase the risks of other diseases and even become life-threatening [32,33]. Indeed, despite continued and incessant efforts to develop a solution for MetS, current drugs have been used for symptomatic treatment [29]. It is well known that healthy diet and regular physical exercise are the most effective interventions to avoid MetS, but once diagnosed, most MetS patients find difficulty returning to those interventions [34,35]. Considering the associations of gut microbiota, which are the companions for humans from birth to human metabolism [36,37,38], it is reasonable to think that gut microbiota can be an emerging medication for MetS and its related diseases. In this review, we will give overview of the current prevention of and treatment for MetS and its related diseases by interpreting the effects of some pharmaceutical drugs considered as an initial choice. In addition, this study will contribute to our understanding of how gut microbiota modulates human metabolisms, exploring the possibility of developing gut microbiota as pharmabiotics in a completely new medication option for treating MetS and its related diseases.

## 2. Current Approach

Metabolic syndrome is a complicated cluster of correlated physiological, biochemical, clinical and metabolic factors that directly increases the risks of CVDs and diabetes [4]. Therefore, treatment of MetS needs to focus on tackling all disorders together. The major goal of treating MetS is to reduce the risk of CVDs including raising low-density lipoprotein cholesterol (LDL-C) and reducing HDL-C, high blood pressure and abdominal fat [4]. Besides, managing blood glucose in diabetes is also mandatory to achieve the goals of therapy in MetS [4]. Current management of MetS depends on pharmaceutical treatments. Based on the current criteria and guidelines for the treatment of patients with MetS, various medical therapies are used to improve obesity, correct lipid profiles and control hypertension and hyperglycemia of patients. In this part, we will discuss the current pharmaceutical treatments of MetS with the drug groups mentioned above (Table 1).

Since obesity is the main predisposing condition behind many of the additional symptoms, closely increasing the risks of CDVs, the priority of clinical treatment in individuals with central obesity and MetS is weight reduction and maintenance of a lower weight [102]. It is well known that lifestyle changes including physical activities and diet modification are recommended as the first line of prevention and/or treatment for obesity [103]. However, maintaining the lifestyle interventions required at least 12 months of restricted calorie diet and 30 min daily for regular exercises [102,104]. Although these interventions are proven to be effective in weight loss, reducing blood pressure and cardiovascular risk factors, even reducing the incidence of new-onset type 2 diabetes mellitus (T2D) for individuals, the consumption of much time to achieve significant improvements in health status is a drawback in modern life for the community [102]. Therefore, currently available drugs for facilitating weight loss are lipase inhibitors, such as Orlistat, and serotonin agonists, such as Lorcaserin. These drugs showed significant efficacy in reducing body weight in the early duration of treatment; however, this beneficial effect has lessened over time, or even most of the patients regained weight if Orlistat was stopped [40,42]. Especially, the efficacy of Lorcaserin on weight loss can disappear after two years according to previous studies [44,48]. Because the reduced body weight effects are limited while many side effects such as severe liver injury or an increased risk of symptomatic hypoglycemia have been documented, these anti-obesity drugs do not seem to persuade the obese to follow drug therapy [40]. Recently, the United States Food and Drug Administration (FDA) approval of the combination of Phentermine and Topiramate (PHEN/TPM) for the treatment of obesity has received increasing research attention [105,106,107]. PHEN/TPM has shown significant efficacy in achieving weight loss and reducing waist circumference, fasting TG and fasting glucose [106,107]. However, because of its new release, long-term safety evaluation of PHEN/TPM is still lacking. Furthermore, the costly and prolonged treatment of obesity also contributes to reduced numbers of patients receiving pharmaceutical products. In addition, many widely-used drugs were removed from the market after a long time because of their serious side effects that raised doubts in patients about long-term complications. One typical example is Sibutramine. Although this drug had revealed its superiority in the treatment of exogenous obesity, the FDA asked the manufacturer to remove this drug from the market because of an elevated risk of cardiovascular issues and strokes in the studied population [108] (Table 1).

In MetS, the presence of dyslipidemic features has been revealed to result in increased development of CVDs and hypertension [4]. Pharmaceutical therapies that lower LDL-C and/or TG and/or raise HDL-C are recommended including statins, proprotein convertase subtilisin/kexin type 9 (PCSK9) inhibitors, cholesterol absorption inhibitors, bile acid sequestrants, fibrates or nicotinic acid. Although these therapies are used for lowering cholesterol levels, each drug class may target different cholesterol indexes. Statins such as Atorvastatin, Simvastatin and Rosuvastatin, PCSK9 inhibitors such as Alirocumab and Evolocumab or bile acid sequestrants such as Cholestyramine, Colestipol and Colesevelam mainly target LDL-C [80,87,92]; whereas fibrates such as Gemfibrozil and Fenofibrate and nicotinic acids such as niacin immediate-release and niacin extended-release seem to be promising in reducing plasma TG level; especially, nicotinic acid also raises HDL-C more than other drugs in dyslipidemic treatment [98,101]. Additionally, in each class, different drugs may show variable efficacy and different pleiotropic effects including effects on other cholesterol indexes. For examples, statins are used for lowering cholesterol levels, as well as prevention of coronary heart diseases, heart attack and stroke, in which Rosuvastatin has the highest efficacy in reducing LDL-C than other statins at comparable doses, while atorvastatin may improve hepatic insulin sensitivity that adds a point in treating patients with both T2D and dyslipidemia [78]. Simvastatin also can potentially increase HDL-C levels, associated with reduced risks of atherosclerosis CVDs [63]. However, besides beneficial effects on correction dyslipidemia, pharmaceutical drugs have various documented side effects [90]. It has been proven that Atorvastatin raised the risk of T2D, induced myopathy with an elevation of creatine kinase and rhabdomyolysis [78,79], while Simvastatin raised T2D risk and the level of blood glucose [84]. Alirocumab induced injection site reactions, which was administered by subcutaneous injection [87], and Ezetimibe might induce rhabdomyolysis [90]. Since bile acid sequestrants caused constipation frequently, they were used cautiously by patients with MetS and hypertension, cerebral diseases and cardiac diseases [92]. Some papers have confirmed that Gemfibrozil enhanced the risk of cancer, while nicotinic acid was associated with liver failure and elevation of blood glucose [97] (Table 1).

T2D is widely considered to be one important component in MetS. Metformin, an insulin-sensitizing agent, is the most commonly-prescribed drug in the world for the treatment T2D [51]. Currently, Metformin is the most effective drug to control glucose level and decreases the risk of new-onset diabetes in MetS patients [51]. Other beneficial effects of Metformin are the prevention of weight gain, reducing the risk of chronic degenerative diseases and even anti-cancer benefits, etc. [109]. Recent studies also suggest the potential of newly identified drugs giving diabetic patients more choices to control blood glucose including the glucagon-like peptide (GLP) agonists, such as Exenatide, and DPP-4 inhibitors, such as Sitagliptin [57,60]. The GLP agonists decrease both endogenous and exogenous sugar by regulating pancreas response, pancreatic release of glucagon in response to eating, gastric emptying and liver fat content [57]; DPP-4 inhibitors elevate insulin secretion and reduce gastric emptying and blood glucose levels by regulating GLP levels, which induce suppression of glucagon release [60]. Because of the special mechanisms of these drugs, each of them targets different patients. Furthermore, evidence of long-term effects of these drugs has not been adequately addressed. Besides this limitation, some of them also have various serious side effects. It has been proven that Thiazolidinediones enhanced the risk of developing or worsening heart failure, developing certain types of cancer and induced bone fractures [52]; sulfonylureas caused cardiovascular mortality, while Meglitinides and Exenatide raised the incidence of benign adenomas of the thyroid and liver [53,54]; Sitagliptin induced renal failure and hypersensitivity reactions [61]. It should be noted that metabolic-related diseases are chronic disorders; therefore, not only those diseases themselves, but also their long-term complications raised difficulties for treatment. Indeed, the burden of diabetes is exacerbated by the prevalence, as well as numerous serious complications including blindness, kidney failure and CVDs [53,54,61]. Obviously, when drugs are used over time such as in the case of diabetes or hypertension patients, drug tolerance can develop, or patients also can become drug-dependent people (Table 1).

Because atherosclerotic CVDs develop significantly as a consequence of diabetes and dyslipidemia, aspirin is used as pharmaceutical intervention in the treatment and prevention of CVDs. Many studies reported that elevated LDL-C levels, reduced HDL-C and raised free fatty acids may all have the potential to increase thrombosis in MetS [4]. Therefore, using aspirin is associated with an increase in the prevalence of ischemic heart disease and stroke due to the atherosclerosis and coagulation disorders involved [4]. Although aspirin’s effects on the prevention of cardiovascular events through platelet aggregation, alterations of coagulation and hemodynamic are demonstrated, using aspirin is still controversial [73]. The range between efficacy and toxicity in aspirin is narrow; thus, adjusting the dose of this therapeutic agent is a challenge for the physician. Daily aspirin therapy can increase stroke risk caused by a burst blood vessel, gastrointestinal bleeding and trigger a serious allergic reaction [72] (Table 1).

Commonly-used drugs for hypertension are angiotensin-converting enzyme (ACE) inhibitors (including Enalapril) and angiotensin-receptor blockers (ARBs) (including Azilsartan, Telmisartan). ACE inhibitors diminish in blood volume, as well as induce relaxation of blood vessels by blocking the conversion of angiotensin I (AI) to angiotensin II (AII) [64]; while ARBs result in vasodilation, decreasing the secretion of vasopressin and aldosterone by regulating the activation of AII type 1 receptors [68]. Both of them are common drugs that are used to regulate blood pressure, but it has been found that Enalapril reduced sleep quality by a dry cough, one of its side effects, which induced tiredness and elevated the level of blood pressure [62]. Both Azilsartan and Telmisartan increased the level of blood potassium, caused kidney failure and increased risk of myocardial infarction [67,69] (Table 1).

Taken together, current drugs present their functions through targeting specific pathways, and therefore, the impacts on multiple abnormalities in MetS and its related diseases have been limited and have been symptomatic treatments. According to the above content, besides the short-term benefits, medications are reported for their limited efficacy, as well as serious side effects in the long-term duration of treatment. The therapy for MetS is a contradiction that confuses the pros and cons of pharmaceutical interventions. Due to the remarkable increase in both the prevalence and severity, as well as the limitations of current pharmaceutical drugs for treating and preventing MetS and its related diseases, the prediction of safe, systemic therapy for MetS is extremely urgent.

## 3. Gut Microbiota Affects MetS and Its Related Diseases

**Figure 1:**
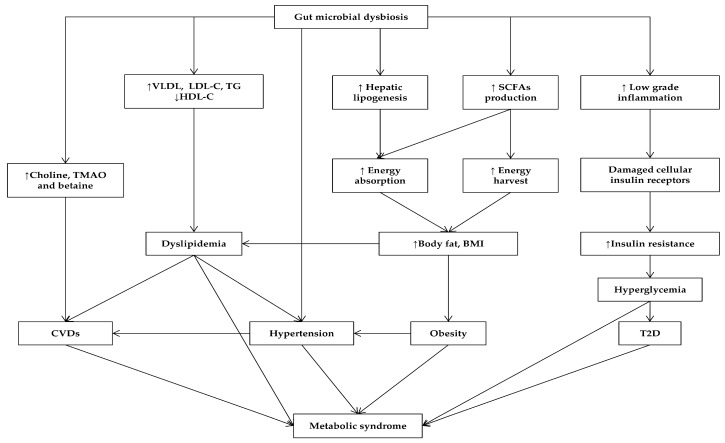
Gut microbiota affects MetS and its related diseases. Arrows indicate increase (↑) or decrease (↓). TMAO, trimethylamine N-oxide.

The human intestine harbors a complex of microbial species collectively known as gut microbiota that plays essential roles in physiology, development and diseases of the host [36,38,110]. The development of culture-independent methods such as metagenomics sequencing, PCR-denaturing gradient gel electrophoresis, microarray and fluorescence in situ hybridization, has expanded our understanding of this diverse microbial community [111]. During birth, the human gut microbial composition begins rapidly and fluctuates until it reaches maturity within the first years of life [112,113]. In general, the predominant gut microbial composition remains relatively stable during the entire lifetime [114]. Establishing the diversity and maintaining the stability and balance of gut microbiota are the key requirements for ensuring human health and well-being [115]. Therefore, the alteration of the diversity or structure of gut microbiota is known as dysbiosis, which may change metabolic activities, which results in metabolic disorders [116] (Figure 1). The remarkable increase in the prevalence and severity of MetS throughout the world recently is stimulating efforts to explore the relationship between gut microbiota and their host metabolism, which mainly relates to the MetS epidemic [116].

### 3.1. Gut Microbiota in Obesity

Numerous studies have deciphered the influences of gut microbiota on host metabolic processes by increasing energy savings from the diet or alternating host metabolic pathways due to microbiota-derived metabolites [37]. Current knowledge has defined that Firmicutes, Bacteroidetes, Actinobacteria and Proteobacteria are the four dominant bacterial phyla in human gut [115]. Considered as a typical function of gut microbiota, fermentation indigestible carbohydrates and plant polysaccharides occur mainly in the large intestine, and thus, approximately 10–30% of the total energy intake would not be eliminated depending on this extraction process of gut microbiota [117,118]. The major fermented end products, short chain fatty acids (SCFAs), mainly acetate, butyrate and propionate, contribute extra energy to the host and thereby directly affect energy regulation [117]. Therefore, the presence of gut microbiota increases SCFAs’ concentration in the colon, as well as minimizes the caloric excretion via the stool [119]. Most studies reported that the gut microbial compositions between obese and lean individuals were significantly different [119,120]. There is a shift in the Firmicutes-Bacteroidetes ratio in which the increased numbers of Firmicutes were accompanied by a decrease in the numbers of Bacteroidetes in obese humans [121]. In addition, the fact that many Firmicutes such as *Eubacterium*, *Faecalibacterium* and *Roseburia* are butyrate producers also supported the high SCFAs’ concentration in obese cecum [122,123]. A more recent study in comparative genomics analysis demonstrated that each bacterium was associated with obesity and weight gain due to containing genes encoded for proteins involved in lipid and carbohydrate metabolism [124]. Indeed, the obese individuals possessed more genes involved in the degradation of complex carbohydrates in the diet as compared to the lean ones [122]. The study of the gut microbiome in adult twin pairs for the lean or obese phenotype is raising the possibility that different physiologic states are determined by an altered contribution of bacterial genes of the core microbiome at a functional level [125]. In addition, the gut microbiota was also associated with diet-induced obesity by two complementary, but independent pathways through the suppression of fasting-induced adipocyte factor (FIAF) and AMP-activated protein kinase (AMPK). The mice colonized with gut microbiota suppressed the intestinal expression of FIAF, a lipoprotein lipase (LPL) inhibitor, whose alteration led to an increase in LPL activity, thereby promoting the cellular uptake of fatty acids and enhancing the accumulation of triglyceride in the adipose tissue [126,127]. Furthermore, the gut microbiota inhibited the expression of AMPK, leading to reduced fatty acid oxidation in skeletal muscle and consequently contributing to an increased adiposity of the host [127]. Obviously, the gut microbiota is the host in energy metabolism. 

### 3.2. Gut Microbiota Affects Lipid Metabolism

Besides the ability of energy harvesting, gut microbiota improves the efficacy of the energy absorption process through promoting the healthy development of intestinal epithelium, which is responsible for nutrient uptake in the gut [36]. Based on this, the gut microbiota can in part modulate energy storage that directly affects the metabolic phenotype of the host [126,128]. Colonization of germ-free (GF) mice with gut microbiota from obese ones led to a higher increase of adiposity than colonization with gut microbiota from the lean ones [122]. It should be noted that SCFAs produced by gut microbiota not only provide extra energy to the host, but also affect the synthesis of cholesterol and lipogenesis via the gut microbiota-liver axis, consequently promoting metabolic diseases [129,130]. Indeed, a recent study demonstrated that SCFAs such as acetate and butyrate could be used as a substrate for the synthesis of cholesterol and fatty acids, including palmitic acid and stearic acid in the liver, thereby elevating serum total cholesterol and triglyceride concentration [129]. Singh et al. showed that SCFAs impact the liver lipids through increasing the expression of stearoyl CoA desaturated-1, an important regulatory enzyme in the homeostasis of fatty acids [130]. Furthermore, the increased lipogenesis in the liver correlated with the increased very low-density lipoprotein (VLDL) production in blood [131]. The associations between gut microbiota and alteration in BMI and blood lipid levels were also reported in a recent population-based clinical study [132]. The lower abundances of the class Mollicutes, families Christensenellaceae and Rikenellaceae and genus *Dehalobacterium* were related to a high BMI, while the family Clostridiaceae/Lachnospiraceae was related to LDL-C levels and the family Pasteurellaceae, genus *Coprococcus* and genus *Collinsella* species *stercoris* were strongly associated with TG levels. Furthermore, the microbial richness with the dominance of the family Christensenellaceae was positively correlated with an increased HDL-C in blood [132,133]. Another recent study also suggested that gut microbiota might promote CVDs through its metabolites formed from dietary phosphatidylcholines such as betaine, choline and trimethylamine N-oxide (TMAO), which are all shown to predict the risk of promoting atherosclerosis [133,134]. It should be noted that dyslipidemia is characterized by abnormal elevation of TG and decreased levels of HDL-C, directly increasing the risk of arteriosclerosis CVDs in MetS. Therefore, the significant contribution of gut microbiota to the variation of blood lipid profile and BMI provides strong evidence for the potential of an approach that alters the gut microbiota to prevent CVDs.

### 3.3. Gut Microbiota in Diabetes

Current knowledge has supported the role of gut microbiota in the pathogenesis of two main types of diabetes mellitus [135,136]. Changes in gut microbial profiles including an increased abundance of Bacteroides and Streptococci and a reduced abundance of *Clostridium* clusters IV and XIVa, possibly through an inflammation-triggering pathways, might contribute to the progression of type 1 diabetes mellitus (T1D), an autoimmune diabetes that is associated with the damage of insulin-producing beta cells in the pancreas [137]. Once diagnosed, the only treatment method for T1D patients is insulin therapy [138]. Because the process of T1D usually begins very early in life, also known as a critical window time to establish and complete the human gut microbial community, therefore, the role of microbiota could be emphasized in preventing the initiation and progression of the T1D process by establishing a healthy gut microbiota after birth [137]. In contrast to T1D, T2D, which is characterized by insulin resistance, more closely relates to MetS. A recent metagenome-wide association study showed that T2D also is associated with a moderate level of gut microbial dysbiosis [139]. The T2D gut microbiota showed an increase in opportunistic pathogens, such as *Bacteroides caccae*, *Clostridium hathewayi*, *Clostridium ramosum*, *Clostridium symbiosum*, *Eggerthella lenta* and *Escherichia coli*, and a reduction of many butyrate-producing bacteria, including *Clostridiales* sp. SS3/4, *Eubacterium rectale*, *Faecalibacterium prausnitzii*, *Roseburia intestinalis* and *Roseburia inulinivorans* [139]. Other studies described the alteration including reduced numbers of *Faecalibacterium prausnitzii* and *Roseburia intestinalis* and an increased number of *Lactobacillus gasseri* and *Streptococcus mutans* found in the gut microbiota community in T2D [140]. Colonization of GF mice with gut microbiota results in accumulation of body fat and increasing insulin resistance [126]. The insulin resistance in T2D is associated with low-grade inflammation occurring in skeletal muscle, adipose tissue or liver, tissues related to metabolic regulation [141]. Insulin resistance is considered as a consequence of the damage of cellular insulin receptors by the effects of excessive expression of pro-inflammatory cytokines, such as interleukin-1, interleukin-6 and tumor necrosis factor alpha [142]. Notably, the alteration in the gut microbial community was associated with an increased concentration of gut microbiota-derived lipopolysaccharide (LPS), commonly known as metabolic endotoxemia, that triggers the expression of the above inflammatory factors via the CD14-dependent mechanism [143,144,145]. As a main LPS receptor, CD14 sets the tone of insulin sensitivity and thus determines the occurrence of metabolic diseases including obesity and diabetes [145]. Cani P.D. and co-workers also revealed that the gut microbiota modulates metabolic endotoxemia and the state of low-grade inflammation by controlling the intestinal permeability that directly affects LPS absorption [146]. Obviously, dysbiosis of the gut microbiota is associated with the pathogenesis of diabetes.

### 3.4. Gut Microbiota and Hypertension

Emerging evidence demonstrated that gut microbiota plays an important role in the development of hypertension, one of the most prevalent cardiovascular diseases, in both animal and human clinical studies [147,148]. In an animal study, high blood pressure is associated with a decrease in the microbial diversity, especially the smaller proportion of Actinobacteria, and the alteration in Firmicutes/Bacteroidetes, with an expansion of the Firmicutes phylum [147]. At the genus level, a decrease in acetate- and butyrate-producing bacteria such as *Coprococcus* and *Pseudobutyrivibrio* and an increase in the lactate-producing bacterial population such as *Streptococcus* and *Turicibacter* were found in the hypertensive model [147]. In humans, through comprehensive metagenomic and metabolomic analyses, gut microbiota dysbiosis was described in the hypertensive phenotype [147]. Compared to healthy controls, the gut microbiota in hypertensive subjects significantly decreased in richness and diversity [148]. A change of microbial composition was found at the genus level with an abundance of *Prevotella* and *Klebsiella* and a declination of *Bifidobacterium*, *Butyrivibrio*, *Coprococcus*, *Faecalibacterium*, *Oscillibacter* and *Roseburia*, which were enriched in healthy controls [148]. Remarkably, transplantation of the gut microbiota from hypertensive donors to GF mice resulted in the appearance of high blood pressure in colonized mice [148]. Collectively, these studies provide important evidence that gut dysbiosis is the key factor for hypertension. Therefore, restoring the homeostasis of gut microbiota could be a potential strategy for treating hypertension and reducing cardiovascular risks.

### 3.5. Gut Microbiota and Psychiatric Problems

In general, accumulating evidence has shown that dietary habits profoundly influence the gut microbiota, thereby affecting host metabolism and even disease risks [149,150]. The alteration of gut microbiota may be a response to the diet in obesity. However, gut microbiota and its metabolites may affect the regulation of the human feeding behavior including feelings of hunger and satiety depending on gut-brain signaling [151]. By linking to neuropeptidergic circuitry in the hypothalamus via several bacterial products, the gut microbiota modulates human eating behavior for both short-term and long-term periods [151]. Current knowledge suggests that there is a bidirectional communication between gut microbiota and the host brain [152]; thus, the influences of gut microbiota on the metabolic-related diseases via the gut-brain axis have become more important because one of the increasing risk factors that contributes to MetS epidemic is psychiatric issues, such as stress, depression, schizophrenia or bipolar disorder [153]. People with psychiatric problems have a predisposition to metabolic syndrome, especially obesity, because of their physical inactivity, as well as antipsychotic drug-induced side effects [154]. Recent studies also reported that some gut bacteria such as *Lactobacillus rhamnosus* and *Bifidobacterium infantis* reduced anxiety-like and depressive-like behaviors [155,156]. Therefore, formatting and maintaining a “healthy” gut microbiota is a key point to ensure the optimal regulation of the host.

## 4. Conclusions and Future Perspectives

MetS is a worldwide epidemic, threatening both developed and developing countries by a remarkable increase in metabolic-related diseases, including obesity, T2D, CVDs and hypertension. Our review supports the point that metabolic-related diseases are usually correlated with imbalances in the gut microbiota. Indeed, the alterations in the profile of the gut microbiota may influence the extraction and storage of energy, serum lipid levels, blood pressure, neuroendocrine cells and immune functions via regulating the metabolism of the host. A recent study of the Human Microbiome Project Consortium about the structure, functions and diversity of the human microbiome also suggested that a “healthy” gut microbiota is variable among individuals, while the metabolic pathways are evenly distributed and prevalent across all healthy individuals, which means the fluctuation of the core microbiota for metabolism mainly contributes to determining the phenotypes of human [115]. Although current metagenomics sequencing analysis tools show strong evidence of general alterations of the gut community underlying human metabolic-related diseases at family and class levels, specific changes at species levels of this community have not been well described because of the extreme diversity of this microbial community, as well as remaining possible sequencing errors. Therefore, the development of a new technology is needed to determine the defined gut microorganisms for treating and preventing MetS and its related diseases. It should be noted that the early establishment of an appropriate gut microbiota in humans seems to be crucial to develop a diverse and balanced microbial community in later life. Therefore, understanding the stability, as well as the different functional profiles of gut microbiota is an important step in enabling predictions of disease states and developing microbiota-based therapeutic options to correct dysbiosis for preventing or treating metabolic-related diseases. It is well known that changes in dietary habits and physical activities are effective solutions for preventing and/or treating MetS; however, this seems to work effectively for individuals, but applying this in the community is still a challenge. On the other hand, the preventions and treatments for metabolic–related diseases that are currently based on pharmaceutical interventions, however, do not provide a solution for MetS and its related diseases. Therefore, considering the strong associations between gut microbiota and the development and pathogenesis of MetS, we propose that developing the core of gut microbiota as pharmabiotics as a completely new medication options that may be easily applied to the community could be a potential prevention or treatment of metabolic diseases and thus contribute to controlling the worldwide MetS epidemic.

## Figures and Tables

**Table 1:** Current pharmaceutical drugs for treating and preventing MetS and its related diseases.

Groups	Drug Classes	Name, Functions	Possible Side Effects	References
Obesity treatment	Lipase inhibitors	**Orlistat** Prevent fats absorption	Steatorrhea, fecal incontinence, frequent or urgent bowel movements, liver injury, acute kidney injury, colon carcinogenesis	[39,40,41,42,43]
	Serotonin agonists	**Lorcaserin** Regulate appetite, mood and endocrine secretion	Upper respiratory tract infection, depression, anxiety, hallucinogenic, cardiac valvulopathy, suicidal ideation, cancer	[44,45,46,47,48]
Hyperglycemia treatment	Insulin-sensitizing agents	**Metformin or** **Thiazolidinediones** Decrease glucose production and increase the insulin sensitivity	Diarrhea, nausea, abdominal pain, hypoglycemia, high blood lactic acid level, edema, weight gain, heart failure, bone fractures, certain types of cancer	[49,50,51,52]
	Insulin secretagogues	**Sulfonylureas or Meglitinides** Increase fusion of insulin granulae, insulin secretion, release from the beta cells in the pancreas	Weight gain, hypoglycemia gastrointestinal upset, headache, hypersensitivity reactions, adenomas of the thyroid and liver, cardiovascular mortality	[53,54]
	The glucagon-like peptide (GLP) agonists	**Exenatide** Suppress pancreatic release of glucagon in response to eating, slow down gastric emptying, decrease the rate, and decrease liver fat content	Gastroesophageal reflux disease, belching, diarrhea, heartburn, indigestion, nausea, vomiting, dizziness, headache, pancreatitis, thyroid cancer	[55,56,57,58]
	DPP-4 inhibitors	**Sitagliptin** Increase insulin secretion and suppress glucagon release by the alpha cells of the pancreas	Nausea, common cold-like symptoms, photosensitivity, hypoglycemia	[59,60,61]
Hypertensive treatment	Angiotensin converting enzyme (ACE) inhibitors	**Enalapril** Decrease blood pressure	Increase serum creatinine, dizziness, low blood pressure, dry cough, airway compressive angioedema	[62,63,64]
	Angiotensin receptor blockers (ARBs)	**Azilsartan or Telmisartan** Decrease blood pressure	Dizziness, headache, hyperkalemia, hypotension, rash, diarrhea, abnormal liver function, muscle cramp, back pain, insomnia, renal impairment, pharyngitis, myocardial infarction, tachycardia, brachycardia.	[65,66,67,68,69]
Preventive cardiovascular treatment	Antiplatelet	**Aspirin** Suppress prostaglandins and thromboxanes production, platelets function	Gastrointestinal bleeding, gastric mucosal erosion, temporary tinnitus, Reye’s syndrome, swelling, headache, kidney injury, cerebral microbleeds, ischemic stroke, intracerebral hemorrhage, Alzheimer’s disease	[70,71,72,73,74]
Dyslipidemia treatment	Statins	**Atorvastatin, Simvastatin or Rosuvastatin** Decrease cholesterol production, increase levels of HDL-C and prevent the events associated with CVDs	T2D, diarrhea, dyspepsia, myalgia, nausea, memory loss, forgetfulness, eczema, muscle cramps, rhabdomyolysis, heartburn, depression, chest pain, jaundice, extreme tiredness, loss of appetite, flu-like symptoms, unusual bleeding or bruising, worse glycemic control, cholestatic hepatitis, hepatic cirrhosis	[75,76,77,78,79,80,81,82,83,84,85,86]
	PCSK9 inhibitors	**Alirocumab or Evolocumab** Decrease LDL-C level 60–70%, prevent early death from cardiovascular disease	Nose irritation, flu-like symptoms, urinary tract infection, diarrhea, bronchitis, muscle pain, soreness, spasms	[87]
	Cholesterol absorption inhibitors	**Ezetimibe** Decrease LDL-C by decreasing cholesterol absorption in the small intestine	Headache, diarrhea, myalgia, hypersensitivity reactions, myopathy, myalgia, rhabdomyolysis	[88,89,90]
	Bile acid sequestrants	**Cholestyramine, Colestipol or Colesevelam** Decrease blood LDL-C, increase HDL-C	Increased TG, transaminase, headache, flatulence, vomiting, diarrhea, dyspepsia, abdominal pain, nausea, myalgia, intestinal obstruction, liver injury, kidney injury	[91,92,93,94,95]
	Fibrates	**Gemfibrozil or Fenofibrate** Decrease elevated LDL-C, total cholesterol, TG, apo B, increase HDL-C	Headache, back pain, nausea, diarrhea, upper respiratory tract infection, gastrointestinal distress, musculoskeletal pain, gallstone, hypokalemia, cancer.	[96,97,98,99]
	Nicotinic acid	**Niacin immediate release (Niacor) or Niacin extended release (Niaspan)** Decrease LDL-C, increase HDL-C in the blood, decrease TG levels by 15–25%	Flushing of the face and neck along with warmth, headache, burning, sweating, chills, dizziness, stomach upset, heartburn, vomiting, diarrhea, indigestion, nausea, liver failure and hyperglycemia	[100,101]
